# Measuring CPT-1-mediated respiration in permeabilized cells and isolated mitochondria

**DOI:** 10.1016/j.xpro.2021.100687

**Published:** 2021-07-31

**Authors:** Krista Yang, Mary T. Doan, Linsey Stiles, Ajit S. Divakaruni

**Affiliations:** 1 Department of Molecular and Medical Pharmacology, UCLA David Geffen School of Medicine, Los Angeles, CA 90095, USA; 2 Department of Medicine, UCLA David Geffen School of Medicine, Los Angeles, CA 90095, USA

**Keywords:** Cell-based Assays, Cell separation/fractionation, Metabolism

## Abstract

Carnitine palmitoyltransferase-1 (CPT-1) is a rate-controlling enzyme for long-chain fatty acid oxidation. This manuscript provides protocols for measuring CPT-1-mediated respiration in permeabilized, adherent cell monolayers and mitochondria freshly isolated from tissue, along with examples to assess the potency and specificity of interventions targeting CPT-1. Strengths of the approach include ease, speed, and breadth of analysis, whereas drawbacks include loss of physiological regulation in reductionist systems and indirect assessment of CPT-1 enzymatic activity.

For complete details on the use and execution of this protocol, please refer to Divakaruni et al. (2018).

## Highlights

• Protocol for pathway-specific respiration using Seahorse XF Analyzer
• Detailed steps for both isolated mitochondria and plasma membrane-permeabilized cells
• Examples given for specific inhibition of long-chain fatty acid oxidation

## Graphical abstract

**Figure:**
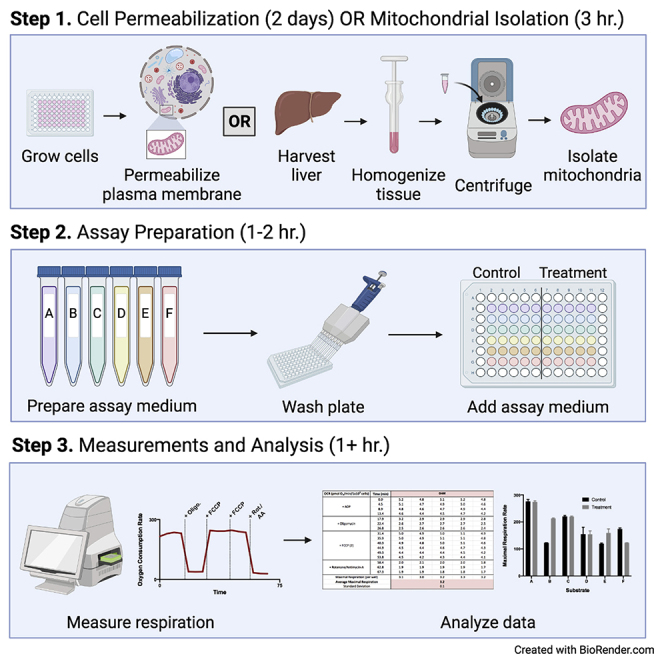

## Before you begin

This protocol is written for use with a Seahorse XF Analyzer, but the approach is broadly adaptable to other plate-based fluorescent or platinum electrode-based techniques to measure oxygen consumption. A full list of critical reagents and consumables is provided in the key resources table. Each section will be split when appropriate to provide specific information for use of this protocol with permeabilized cells or isolated mitochondria. Cultured hepatocytes and isolated liver mitochondria are used as examples, but this analysis can be extended to any cell or tissue type with suitable rates of oxidation for the substrate(s) of interest (e.g., long-chain fatty acids). We present both protocols to provide as broad of a resource as possible: when starting with cultured cells it is almost always preferable to work with permeabilized cells over isolated mitochondria, whereas working with animal tissue often lends itself to isolating mitochondria.

This protocol was used previously to determine the on-target concentration of the CPT-1 inhibitor etomoxir and its off-target effects on oxidative phosphorylation (Divakaruni et al., 2018). Prior to using this protocol, researchers should familiarize themselves with a standardized, intact cell respirometry assay with the Seahorse XF Analyzer (Gotoh et al., 2021; Gu et al., 2021; Pelletier et al., 2014).

All Seahorse XF experiments require the measurement cartridge to be hydrated in proprietary calibrant for 8–48 h (usually overnight) prior to the assay. Hydrate the Seahorse XF cartridge (from the Extracellular Flux Assay Kit) by soaking fluorophores in 200 μL of manufacturer-supplied XF calibrant per well according to the manufacturer’s instructions. The cartridge should be incubated at 37°C in a humidified, non-CO_2_ incubator overnight (i.e., 8–20 h). This step takes 5–10 min on the day prior to the assay.

### Permeabilized cells

To permeabilize the plasma membrane prior to measurements, intact cells are treated with recombinant perfingolysin O (rPFO), a cholesterol-dependent cytolysin (Heuck et al., 2010; Schulz, 1990). This creates pores in the plasma membrane, diluting the cytoplasmic contents with the experimental medium and allowing experimental control of substrate provision to the mitochondria (Divakaruni et al., 2013).

The cell density for seeding into Seahorse XF cell culture plates must be optimized prior to analysis (Pelletier et al., 2014). Cells should be maintained according to supplier instructions [e.g., American Type Culture Collection (ATCC)]. The outer rim of the plate should be filled with PBS or medium to avoid evaporative or temperature-based effects across the microplate (Lundholt, 2003).

### Cell culture

**Timing: 20–30 min, 2 (or more) days prior to the experiment**

1. Culture and prepare cells according to suppliers’ recommendations. HepG2 cells (ATCC HB-8065) are presented here as an example, though this protocol can be adapted to almost every cell type.
2. Two days prior to the assay, harvest and plate cells at the desired density in the inner 60 wells of Seahorse XF96 well plates. For uniform plating, ensure the cells are well-mixed in the basin prior to each plating event by pipetting the cell mixture up and down multiple times.
  a. HepG2 cells, maintained below 10 cell passages, are plated in 100 μL at 2.5 × 10^4^ cells/well. For this density, cells are resuspended at a stock concentration of 2.5 × 10^5^ cells/mL and 100 μL is plated into each well using a multichannel pipette.

**CRITICAL:** Researchers should optimize cell density depending on the unique growth rate and assay conditions for each experiment.

3. Fill the outer wells of the plate with 200 μL of PBS or medium to guard against evaporative or temperature-based effects. This can also be done prior to plating the cells.

***Note:*** We recommend running no more than 12 experimental groups for each assay, with no less than 5 technical replicates per group. This can be done using the inner 60 wells of the plate, obviating the need to use any wells along the edge of the plate that are subject to evaporative or temperature gradients (Lundholt, 2003).

***Note:*** Nonadherent cells (i.e., cells grown in suspension) can be harvested, counted, and immediately plated prior to the assay. Cells should be adhered to the XF cell culture plate using Cell-Tak according to manufacturer’s instructions. Treating the plate with Cell-Tak will require 1 h on the day of the experiment to treat, wash, and dry the assay plate.

### Isolated mitochondria

Mitochondria from rodent tissue are freshly isolated on the day of the assay, though it is advised to make necessary buffers and reagent stocks on the day(s) prior to the assay. On the day of the assay, bovine serum albumin (BSA) should be added to any buffers and the pH checked/adjusted prior to use.

### Mitochondrial isolation from mouse liver

**Timing: 2–3 h, morning of the experiment**

**CRITICAL:** Mitochondrial isolations are different for each tissue and vary widely regarding methods of tissue disruption, homogenization specifics, centrifugation speeds, etc. This protocol is adapted from Rogers et al. (2011). It is specific to rodent liver mitochondria and should not be applied to other tissues. Videos of a broadly similar mitochondrial isolation from skeletal muscle mitochondria can be viewed for further guidance (Djafarzadeh and Jakob, 2017).

**Figure 1:**
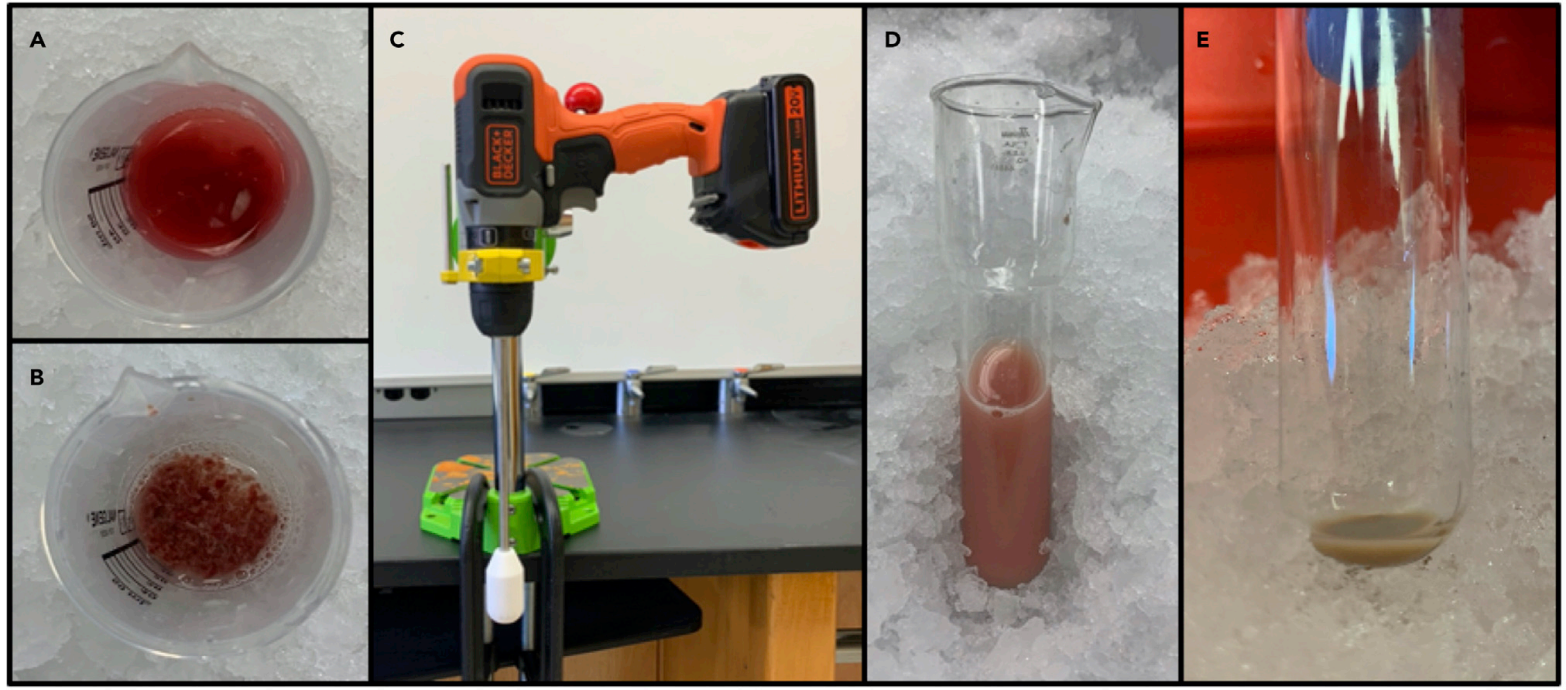
Isolation of mouse liver mitochondria (A) Liver partially minced with scissors. The buffer is cloudy and opaque with blood. It should be continuously drained and replaced with new buffer during mincing. (B) Liver fully minced with scissors. Cloudy buffer has been repeatedly drained and replaced with new buffer. (C) Sample drill-press homogenizer set-up. (D) Fully homogenized liver tissue following drill-press homogenization. (E) Final pellet with both supernatant and light, ‘fluffy’ layer removed.

4. Prior to sample collection, set centrifuges to 4°C. Pre-chill homogenizers, glassware and plasticware, buffers, and tubes on ice. Ensure everything remains as cold as possible throughout the isolation procedure.
5. Euthanize the mouse with isoflurane followed by cervical dislocation in accordance with institutional IACUC guidelines. Spray the carcass with ethanol to mat the fur, and use scissors to make a U-shaped incision in the lower abdominal area to open the peritoneal wall. Extract the liver with forceps/scissors as quickly as possible and place into a 50 mL beaker with just enough ice-cold MSHE (+BSA) to cover the tissue. Mice of both sexes can be used for mitochondrial isolation at any age 4 weeks and older depending on the experimental details (e.g., aging study). The animals presented here are males between 8–10 weeks.
6. Quickly mince the liver with scissors while keeping the beaker submerged in ice (Figure 1A). At frequent intervals during mixing, let the tissue settle and pour off the medium containing blood, fat, and any connective tissue. Add fresh MSHE (+BSA) and repeat this process of mincing and draining until the tissue is finely minced and the medium is clear (Figure 1B).

**CRITICAL:** Steps 5 and 6 should be conducted as quickly as possible to avoid hypoxic damage to the tissue.

7. Transfer the liver tissue into the pre-chilled 15 mL Teflon glass homogenizer receptacle, using MSHE (+BSA) to wash out any remaining pieces. Fill the homogenizer with MSHE (+BSA) to approximately three-fourths of the lower, narrow part of the receptacle.

***Note:*** If using livers from multiple mice or a large rat, scale up to a 40 mL homogenizer.

8. Use a drill-driven Teflon pestle (Figure 1C) to disrupt the tissue in 2–3 strokes. The mixture should be homogenous and free of debris (Figure 1D). Transfer the mixture into two pre-chilled centrifuge tubes and fill to approximately four-fifths volume with MSHE (+BSA).
9. Centrifuge at 1000 *g* for 3 min at 4°C.
10. While spinning, use MSHE (+BSA) to wet two layers of muslin or cheesecloth and place them over the mouth of a clean, pre-chilled conical tube.
11. Decant the supernatant through the wet cheesecloth to filter, taking care to not spill. Divide the filtered supernatant into two pre-chilled centrifuge tubes and spin at 11,600 *g* for 10 min at 4°C.

***Note:*** If cheesecloth or muslin is unavailable, take extreme care to avoid contaminating the supernatant with the loose pellet while decanting. One may also choose to pipet the supernatant through a 70 μm cell strainer if cheesecloth is unavailable.

12. Aspirate the supernatant and resuspend the pellets in a minimal amount (i.e., 50–100 μL) of MSHE (+ BSA). Combine the resuspended pellets into a single tube and centrifuge along with a balance tube at 11,600 *g* for 10 min at 4°C.
13. Aspirate the supernatant and remove as much of the light, ‘fluffy’ edge of the dark brown pellet as possible (Figure 1E). Resuspend the pellet in as little MSHE *without* BSA as possible (generally 20 μL–100 μL based on the size of the pellet) and keep on ice. BSA should not be used in the resuspension as it will alter the protein measurements. If kept very concentrated and on ice, the mitochondria remain viable for hours after isolation.
14. Measure mitochondrial protein levels using a standard protein assay (e.g., Bradford, BCA, etc.). The yield will depend on several factors (e.g., amount of starting tissue, thoroughness of homogenization, amount of light layer removed, etc.) but, in general, a preparation from a 8–10 week-old mouse yields 15–30 mg of liver mitochondrial protein.

## Key resources table

**Table:** 

REAGENT or RESOURCE	SOURCE	IDENTIFIER
**Chemicals, peptides, and recombinant proteins**		

Etomoxir	Sigma-Aldrich	Cat#E1905; CAS: 828934-41-4
Oligomycin	Sigma-Aldrich	Cat# 75351;CAS: 579-13-5
Rotenone	Sigma-Aldrich	Cat#R8875; CAS: 83-79-4
Antimycin A	Sigma-Aldrich	Cat#A8674; CAS: 1397-94-0
Carbonyl cyanide 4-(trifluoromethoxy)phenylhydrazone (FCCP)	Sigma-Aldrich	Cat# C2920; CAS: 370-86-5
Coenzyme A	Sigma-Aldrich	Cat#C4780; CAS: 55672-92-9
Adenosine 5′-disphosphate (ADP)	Sigma-Aldrich	Cat#A5285; CAS: 72696-48-1
Pyruvic acid	Sigma-Aldrich	Cat#107360; CAS: 127-17-3
Malic acid	Sigma-Aldrich	Cat#240176; CAS: 6915-15-7
Palmitoyl coenzyme A (Palmitoyl CoA)	Sigma-Aldrich	Cat#P9716; CAS: 188174-64-3
Palmitoyl carnitine	Sigma-Aldrich	Cat#61251; CAS: 2364-67-2
Glutamic acid	Sigma-Aldrich	Cat#G1251; CAS: 56-86-0
Succinic acid	Sigma-Aldrich	Cat#S3674; CAS: 110-15-6
Seahorse XF Plasma Membrane Permeabilizer	Agilent	Cat#102504-100
Minimum Essential Medium (MEM)	Fisher Scientific	Cat#11-095-098
GlutaMAX	Fisher Scientific	Cat#35-050-061
Penicillin-Streptomycin	Fisher Scientific	Cat#15-140-122
Carnitine	Bachem	Cat#F-2700; CAS: 541-15-1
HyClone Fetal Bovine Serum (FBS)	Fisher Scientific	Cat #SH3007103
Mannitol	Sigma-Aldrich	Cat#M9546; CAS: 69-65-8
Sucrose	Sigma-Aldrich	Cat#84097; CAS: 57-50-1
HEPES buffer	Sigma-Aldrich	Cat#83264; CAS: 7365-45-9
Egtazic acid (EGTA)	Sigma-Aldrich	Cat#E3889; CAS: 67-42-5
Bovine serum albumin (BSA)	Sigma-Aldrich	Cat#2905; CAS: 9048-46-8
Potassium hydroxide	Sigma-Aldrich	Cat#221473; CAS: 1310-58-3
Sterile-filtered water	Sigma-Aldrich	Cat#W3500; CAS: 7732-18-5
Potassim phosphate monobasic	Sigma-Aldrich	Cat#P5655; CAS: 7778-77-0
Magnesium chloride solution (1M)	Sigma-Aldrich	Cat#63069; CAS: 7786-30-3
Dulbecco's phosphate-buffered saline (DPBS)	Fisher Scientific	Cat#14-190-250
16% Paraformaldehyde	Fisher Scientific	Cat#50-980-487
Hoescht 33342	Fisher Scientific	Cat#H3570; CAS: 875756-97-1
Cell-Tak	Fisher Scientific	Cat#CB-40240
Calcium chloride solution	Sigma-Aldrich	Cat#21115; CAS: 10043-52-4
Glycerol 3-phosphpate (G3P)	Sigma-Aldrich	Cat#G7886; CAS: 29849-82-9
Ascorbic acid	Sigma-Aldrich	Cat#A5960; CAS: 50-81-7
TMPD	Sigma-Aldrich	Cat#87890; CAS:637-01-4

**Critical commercial assays**		

Seahorse XF FluxPak (cartridges, microplates, calibrant)	Agilent	Cat#102416-100

**Experimental models: Cell lines**		

HepG2	ATCC	Cat#HB-8065; RRID: CVCL_0027

**Experimental models: Organisms/strains**		

C57/BL6J mouse (male, 8–10 weeks age)	The Jackson Laboratory	Cat#000664

**Software and algorithms**		

Agilent Seahorse XF WAVE	Agilent	http://www.agilent.com/en/promotions/
Microsoft Excel	Microsoft	https://www.microsoft.com/en-us/microsoft-365/excel
Prism 9 GraphPad	GraphPad	https://www.graphpad.com/

**Other**		

Cheesecloth	Fisher Scientific	Cat#06-665-28
Tissue grinder	Fisher Scientific	Ca#NC0535680
Sterile vacuum filtration system	Sigma-Aldrich	Cat#S2GPU05RE
Seahorse XFe96 Analyzer	Agilent	N/A
Operetta CLS High-Content Analysis System	PerkinElmer	N/A
Multimode plate reader	Tecan	N/A

## Materials and equipment

**Table:** 1× MAS buffer *without* BSA (can be made prior to the assay and stored at 4°C for 3 months)

Reagent	Final concentration	Amount
Mannitol	220 mM	20.04 g
Sucrose	70 mM	11.98 g
Potassium Monophosphate (KH_2_PO_4_)	10 mM	0.68 g
Magnesium Chloride (MgCl_2_, 1M)	5 mM	2.5 mL
EGTA (250mM stock solution; pH to 8.0 with KOH)	1 mM	2 mL
HEPES (1M)	2 mM	1 mL
KOH (1M)	n/a	to pH 7.2
Tissue culture grade water	n/a	fill to 500 mL
**Total**	**n/a**	**500 mL**

***Note:*** If storing 1× medium, pH to 7.2 at 4°C and filter sterilize using a 0.22 micron filter. The MAS without BSA can also be made as a 3× stock solution to dilute to 1× on the day of the assay. Precipitates will form in 3× MAS if kept at 4°C for extended periods, which can be avoided by not pH’ing the 3× stock solution.

**Table:** MAS with 0.2% (w/v) BSA

Reagent	Final concentration	Amount
MAS without BSA	n/a	98 mL
Fatty acid-free (Fraction V) BSA (10% w/v sterile stock solution)	0.2% (w/v)	2 mL
**Total**	**n/a**	**100 mL**

***Note:*** The BSA should be added to the MAS on the day of the assay for sterility.

**Table:** Stock solutions of mitochondrial effectors

Reagent	Final concentration	Amount
Oligomycin	6.3 mM in 95% EtOH	10 mg in 2 mL EtOH
FCCP	10 mM in 95% EtOH	10 mg in 3.93 mL EtOH
Rotenone	2 mM in 95% EtOH	10 mg in 12.68 mL EtOH
Antimycin A	10 mM in 95% EtOH	10 mg in 1.71 mL EtOH

***Note:*** Stock solutions for mitochondrial effectors should be apportioned into 250 μL aliquots and can be stored at −20°C for 6 months.

**CRITICAL:** Oligomycin, rotenone, and antimycin A may be harmful by irritation or skin absorption. Hands should be washed thoroughly after use and personal protective equipment should be worn at all times.

**Table:** Stock solutions of mitochondrial substrates and cofactors

Reagent	Final concentration	Amount
ADP	0.5M, pH 7.2 (with KOH)	1 g in 3.99 mL (final volume) TC-grade H_2_O
BSA	10% (w/v)	1 g in 10 mL (final volume) TC-grade H_2_O
Pyruvate	0.5M, pH 7.2 (with KOH)	1 g in 22.71 mL (final volume) TC-grade H_2_O
Malate	0.5M, pH 7.2 (with KOH)	1 g in 14.92 mL (final volume) TC-grade H_2_O
Glutamate	0.5M, pH 7.2 (with KOH)	1 g in 13.59 mL (final volume) TC-grade H_2_O
Palmitoyl CoA	10 mM	10 mg in 1 mL TC-grade H_2_O
Carnitine	0.5M, pH 7.2 (with KOH)	1 g in 12.41 mL (final volume) TC-grade H_2_O
Palmitoyl carnitine	10 mM	10 mg in 2.50 mL 95% EtOH
Succinate	0.5M, pH 7.2 (with KOH)	1 g in 16.94 mL (final volume) TC-grade H_2_O
Glycerol-3-phosphate	0.25M, pH 7.2 (with KOH)	1 g in 10.80 mL (final volume) TC-grade H_2_O
Ascorbate	1M, pH 7.2 (with KOH)	1 g in 5.68 mL (final volume) TC-grade H_2_O
TMPD	10 mM	1 g in 4.22 mL (final volume) TC-grade H_2_O

***Note:*** Stock solutions for mitochondrial substrates and co-factors should be apportioned into 250 μL aliquots and can be stored at −20°C for 6 months.

### Materials specific to permeabilized cells

**Table:** HepG2 Culture Medium (can be made prior to the assay and stored at 4°C for 1 month)

Reagent	Final concentration	Amount
GlutaMAX	1:100	5 mL
Penicillin/Streptomycin	1:100	5 mL
Fetal Bovine Serum	10%	50 mL
Minimum Essential Medium	n/a	440 mL
**Total**	**n/a**	**500 mL**

### Materials specific to isolated mitochondria

**Table:** MSHE without BSA (can be made and stored ahead of time at 4°C for 3 months)

Reagent	Final concentration	Amount
Mannitol	210 mM	19.13 g
Sucrose	70 mM	11.98 g
HEPES (1M stock solution)	5 mM	2.5 mL
EGTA (250 mM stock solution; pH to 8.0 with KOH)	1 mM	2 mL
KOH (1 and 10M stock solutions)	n/a	to pH 7.2
Tissue culture grade water	n/a	fill to 500 mL
**Total**	**n/a**	**500 mL**

***Note:*** If storing buffer, pH to 7.2 at 4°C and filter sterilize using a 0.22 micron filter. The MSHE without BSA can also be made as a 3× stock solution to dilute to 1× on the day of the assay.

**Table:** MSHE with 0.2% (w/v) BSA

Reagent	Final concentration	Amount
MSHE without BSA	1×	245 mL
Fatty acid-free (Fraction V) BSA (10% w/v sterile stock solution)	0.2% (w/v)	5 mL
**Total**	**n/a**	**500 mL**

***Note:*** The BSA should be added to the MSHE on the day of the assay for the purposes of sterility.

All other reagents are listed in the key resources table.

### Equipment

#### Seahorse XF Analyzer

Oxygen consumption rates are measured using a Seahorse XF Analyzer.

***Alternatives:*** If a Seahorse instrument is unavailable, similar measurements can be made using fluorescent dyes, such as Luxcell MitoXpress, with a standard 96-well plate reader (Will et al., 2007). A platinum-based, Clark-type oxygen electrode could also be used, though the number of groups required for the examples presented (concentration-response curves or analysis of multiple substrates) makes this practically challenging (Doerrier et al., 2018).

### Equipment specific to permeabilized cells

#### High-content imaging system for normalization to nuclei

A high-content imaging system is preferred for normalizing rates of oxygen consumption to cell number. In this protocol, *post hoc* cell number was assessed in paraformaldehyde-fixed plates using the Perkin-Elmer Operetta to count Hoechst-stained nuclei.

***Alternatives:*** Most high content imaging systems (e.g., Bio-Tek Cytation) can be adapted for Seahorse XF96 plates. If such a system is unavailable, bulk fluorescence with a nuclear stain or measurement of total protein can be used, though these methods should be validated to ensure they can sufficiently detect subtle changes in cell number.

### Equipment specific to isolated mitochondria

#### Dounce homogenizer or tissue disruptor

For isolating mitochondria from liver, a drill-driven serrated Teflon pestle in a glass homogenizer with a pouring spout is advised. Other equipment such as a hand-held tissue homogenizer (e.g., Polytron) may be required for isolating mitochondria from heart or skeletal muscle.

***Alternatives:*** A drill press is not explicitly required, as homogenizing tissue may be done by hand, but the yield and quality of the mitochondrial isolation will vary considerably.

#### 96-well plate reader

Absorbance measurements are required for protein determination of isolated mitochondria. This is most convenient in a multi-well plate format but can be accomplished with cuvette-based detection methods as well.

## Step-by-step method details

### Prepare injectates, load and calibrate cartridge

**Timing: 45 min–1 h**

This section covers the preparation of injectates and setup of the Seahorse XF assay. If unfamiliar with the Seahorse XF Assay, the researcher is encouraged to familiarize themselves with the basic setup of the instrument and assay (Gotoh et al., 2021; Gu et al., 2021; Pelletier et al., 2014). This process is shared between both the permeabilized cell and isolated mitochondria assays.

1. Warm 1× MAS medium in a 37°C water bath.
2. Prepare injectates as shown below, adding stock concentrations of mitochondrial effectors [all made in 95% (v/v) EtOH] to plain 1× MAS medium without BSA. Vortex to mix.
  a. No substrates are added to the injectate medium because most assays will involve testing the effects from multiple substrates, and thus 1× MAS is a shared, ‘base’ medium compatible with all conditions. BSA is omitted to avoid potential injection failures from increased medium viscosity.
  b. Sample calculations are presented for one assay plate:

**Table:** 

Port	Stock solution	Final concentration (mM or μM)	Media volume	Effector volume to add
A	Oligomycin (6.3 mM)	2.25 μM	3 mL	7.5 μL
B & C	FCCP (10 mM)	1.5 μM	6 mL	7.2 μL
D	Rotenone (2 mM)	0.2 μM	3 mL	3 μL
	Antimycin-A (10 mM)	1 μM		3 μL

3. Pipette 25 μL of each injectate solution into the corresponding port(s), using a multichannel pipette with the manufacturer-provided loading guides if desired.
  a. Take care to pipet gently but confidently. Avoid too much force so injectates do not escape through the port aperture, but ensure the liquid makes a full seal covering the opening of the port.
  b. The user should familiarize themselves with the dilutions and concentrations required for sequential injections during an XF assay (Pelletier et al., 2014). This assay will be run with an initial volume of 150 μL and sequential 25 μL injections.

***Note:*** In assays where all four ports are available for mitochondrial effects (i.e. no acute injection of test compounds), we suggest FCCP be injected twice to best measure maximal respiration. The concentration of FCCP required to achieve maximal respiration may change between measurement groups (e.g. pyruvate/malate vs. succinate/rotenone, WT vs. KO, etc.).

4. Warm the utility plate and cartridge in a non-CO_2_, 37°C benchtop incubator. While the cartridge is warming, design the plate map and protocol on the Seahorse controller’s Wave application or load a previously made template.
  a. Group Definitions: Define the injections, pre-treatments, assay medium, and cell/mitochondria types.
  b. Plate Map: Label each well with the respective conditions, ensuring to also define the background wells. When plating medium or saline is used in the outer wells during the cell culture (Before you begin: step 3), these wells should be used as background.
  c. Protocol: For permeabilized cells, we suggest a Mix/Wait/Measure (in min:s) cycle of 1:30 Mix/0:00 Wait/2:30 Measure. For isolated mitochondria, we suggest a cycle of 1:30 Mix/0:00 Wait/2:00 Measure. The reduced times [relative to those suggested for intact cells (Pelletier et al., 2014)] presented here are to (i) prevent permeabilized cells from detaching off of the measurement plate over the course of a long assay, and (ii) prevent anoxia in the measurement chamber from depletion of oxygen, particularly with isolated mitochondria.
  d. Save the template if desired and click “Run Assay” to load the cartridge in the instrument for calibration.

***Note:*** It is acceptable to uncheck the “Equilibrate” button, eliminating the Mix/Wait cycle used to ensure the plate is at 37°C. In this case, increase the number of initial measurements (e.g., from three to six) to provide ample time for the plate to warm while also obtaining these measurements.

5. When the instrument opens, **REMOVE THE LID** from the cartridge and place the cartridge and utility plate in the thermal tray with the letters right-side up, aligned on the left of the plate. Start calibration.
  a. Failure to remove the lid can result in major damage to the instrument.

### Seahorse-XF96 assay

**Timing: 1.5–2 h**

This section details the preparation of both permeabilized cells as well as isolated mitochondria for the Seahorse XF Assay. The assay preparations are slightly different but share the same principles. Stocks of reagents and mitochondrial effectors should be made well in advance of the assay. Single-use aliquots can be stored at −20°C and used for several months. All reagents should be prepared in tissue culture-grade H_2_O and used at pH 7.2, adjusted using KOH. Steps 6–10 are given in reference to permeabilized cells, and steps 11–16 for isolated mitochondria. Stocks for substrates, co-substrates, and additives for the experiments are shared between the two methods [apart from the recombinant perfringolysin O (rPFO)], and are presented here:

**Table:** 

	Substrates, co-substrates, and additives (stock concentration)	Final concentration	Volume to add to 1 mL assay media
ADP	ADP (0.5M), pH 7.2 in H_2_O	4 mM	8 μL
Fatty-acid free BSA	BSA [10% (w/v)] in H_2_O	0.2% (w/v)	20 μL
rPFO	rPFO; commercially XF PMP (10 μM)	1–3 nM	0.1–0.3 μL
NADH-linked (Complex I) substrates	Pyruvate (0.5M), pH 7.2 in H_2_O Malate (0.5M), pH 7.2 in H_2_O	5 mM 0.5 mM	10 μL 1 μL
	Glutamate (0.5M), pH 7.2 in H_2_O Malate (0.5M), pH 7.2 in H_2_O	5 mM 5 mM	10 μL 10 μL
	Palmitoyl CoA (10 mM) in H_2_O Malate (0.5M) in H_2_O Carnitine (0.5M) in H_2_O	40 μM 0.5 mM 0.5 mM	4 μL 1 μL 1 μL
	Palmitoyl carnitine (10 mM) in EtOH Malate (0.5M)	40 μM 0.5 mM	4 μL 1 μL
Q-linked (Complex II or III) substrates	Succinate (0.5M), pH 7.2 in H_2_O Rotenone (2 mM) in EtOH	5 mM 2 μM	10 μL 1 μL
	Glycerol-3-phosphate (0.25M), pH 7.2 in H_2_O Rotenone (2 mM) in EtOH CaCl_2_ (1M) in H_2_O	5 mM 2 μM 700 μM total	20 μL 1 μL 0.7 μL
Complex IV substrates	Ascorbate (1M), pH 7.2 in H_2_O TMPD (10 mM) Antimycin A (2 mM)	10 mM 100 μM 1 μM	10 μL 1 μL 0.5 μL

***Note:*** The free calcium concentration with 700 μM CaCl_2_ added to 1 mM EGTA in MAS buffer (pH 7.2, 37°C, 330mOsm/L) is estimated to be 680 nM (Schoenmakers et al., 1992).

**CRITICAL:** It is recommended that ascorbate and TMPD solutions be freshly made on the day of the assay. If stored, keep TMPD solutions shielded from light.

### Permeabilized cells

6. Prepare the assay medium. For permeabilized cells, this almost always includes 1× MAS supplemented with ADP, BSA, permeabilization reagent (rPFO in this protocol), and the appropriate oxidizable substrates. Budget 1 mL of assay medium for every 5 experimental wells.
  a. For example, if 10 wells are being used for one experimental group, prepare 2 mL of assay medium for this group to account for dead volume in the pipette or spills.
  b. If testing the acute effects of pharmacologic compounds, BSA will likely bind hydrophobic compounds and affect the free concentration. BSA can be reduced, or removed, accordingly.
7. Wash the Seahorse XF cell plate with unsupplemented 1× MAS medium. To do so, remove the remaining medium from the inner 60 wells with a multichannel pipette or gentle aspiration, keeping a small meniscus to not leave cells completely dry (i.e., remove 80 μL from the well to leave roughly 20 μL if cells were previously plated in 100 μL). Then add 130 μL of 1× MAS medium. Repeat this process of removing all but about 20 μL of medium and replacing with 130 μL two more times.
  a. It is essential to avoid touching the bottom of the well with the pipet tips or aspirating the cells with too much force, as this will disrupt the cell monolayer. Given the unique geometry of the Seahorse XF plate wells, it is advised to practice with a blank plate ahead of time to get a feel for the process.
8. Use an aspirator or multichannel pipette to completely remove the medium from background wells around the rim and replace with 150 μL of unsupplemented MAS.
9. For the final wash and addition of assay medium, remove all but about 20 μL of medium from the plate (including background wells). Add 130 μL of assay medium with substrates (e.g., pyruvate with malate) to the desired groups and the background wells.
  a. It is important to add assay medium to the background wells during the assay (as opposed to plain MAS) to help with troubleshooting assays. Reasons for this include having the ability to check whether compounds were pH’d appropriately and whether uncharacterized compounds could interfere with the fluorometric measurement probes.
10. Warm the plate in a benchtop, non-CO_2_ incubator set to 37°C for 5 min. When the calibration of the XF cartridge has finished, replace the ejected utility plate with the cell plate (**AFTER REMOVING THE LID)** and continue the run.
  a. Failure to remove the plate lid can result in major damage to the instrument. Do not leave the instrument until verifying that it is running properly.
  b. Provided the measurements used for analysis are conducted at 37°C, we suggest a shorter warming period for permeabilized cell assays than that which is suggested for intact cells for two reasons:
    i. A shorter incubation time prevents permeabilized cells from detaching from the measurement plate over the course of a long assay.
    ii. Since glycolysis is non-functional in a permeabilized cell, ECAR is usually not measured and largely used only for troubleshooting (i.e., ensuring medium alkalinization during ATP synthesis, etc.). As such, there is much less need to worry about the CO_2_ stored in the plastic assay plate during cell culture affecting the pH measurements, obviating this long incubation period mostly used to ‘de-gas’ the plate.

### Isolated mitochondria

11. Prepare the assay medium. For isolated mitochondria, this almost always includes 1× MAS supplemented with ADP, BSA, and the appropriate oxidizable substrates. Budget for 1 mL of assay medium for every 5 experimental wells.
  a. For example, if 10 wells are being used for one experimental group, prepare 2 mL of assay medium for this group to account for dead volume in the pipette or accidents.
  b. Additionally, for each plate, prepare 3 mL of MAS supplemented with ADP and BSA and chill on ice. This will be used in step 12.
12. Place a Seahorse XF plate on ice as well as a basin to be used later. Dilute mitochondria to the appropriate concentration per well using the 3 mL of medium set aside in step 11.
  a. For example, if the mitochondrial stock is 60 mg/mL of mitochondrial protein, then adding 7.5 μL of the mitochondrial stock to 3 mL of medium will result in a final concentration of 0.15 mg/mL. 20 μL of this solution added per well will yield 3 μg/well.
  b. The amount of mitochondria required per well will depend on several factors such as the tissue of isolation, the substrate offered, and the method used to determine the protein concentration. The protein concentration should be optimized to give a robust signal without depleting the oxygen during the measurement period. In our experience with mouse liver mitochondria using the BCA protein assay, the following concentrations are used: 3 μg/well with complex I-linked substrates; 1.5 μg/well with succinate/rotenone; and 0.75 μg/well with ascorbate/TMPD.
13. Gently mix the diluted mitochondrial solution by pipetting up and down with a serological pipet (do not vortex). Empty contents of the tube into the chilled basin (on ice) and plate into the chilled Seahorse plate (on ice). Use a multichannel pipette to plate 20 μL of the mitochondrial solution into each well. Leave at least four wells (usually the corner wells) to be used as background wells, and fill these with 20 μL of plain medium.
14. Centrifuge the plate in a swinging bucket rotor centrifuge for 20 min at 2000 *g.* Adjust the settings of the instrument such that there is no acceleration or brake.
  a. The brake can be set at “1 of 10” on certain instruments provided it is sufficiently slow to not disturb the mitochondrial layer.
15. When finished, return the plate to ice and add 130 μL of assay medium with substrates to the desired groups and the background wells.
  a. It is important to add assay medium to the background wells (as opposed to plain MAS) to help with troubleshooting assays. Reasons include having the ability to check whether compounds were pH’d appropriately and whether uncharacterized compounds could interfere with the fluorometric measurement probes.
16. Warm the plate in a benchtop, non-CO_2_ incubator set to 37°C for 5 min. When calibration has finished, replace the ejected utility plate with the cell plate (**AFTER REMOVING THE LID)** and continue the run.
  a. Failure to remove the plate lid can result in major damage to the instrument. Do not leave the instrument until verifying that it is running properly.

### Normalization

**Timing: 2–2.5 h**

### Permeabilized cells

This section covers the fixing, staining, and counting of cell nuclei in the permeabilized cell assay. No *post hoc* normalization is required for experiments with isolated mitochondria, as this data is normalized to the amount loaded in the plate (e.g., pmol O_2_/min/μg mitochondrial protein)

17. Once the permeabilized cell run has completed, eject the plate and remove 175 μL of medium from inner 60 wells using a multichannel pipette. In a chemical fume hood, add 25 μL of 16% (v/v) paraformaldehyde, followed by 100 μL PBS for a final volume of 200 μL. Let the plate rest for at least 30 min.
  a. It is essential to avoid touching the bottom of the well with the pipet tips or aspirating the cells with too much force, as this will disrupt the cell monolayer.
  b. After this step, the edges of the plate can be sealed with parafilm and stored at 4°C for several days until further use.
18. When ready to stain, make 7 mL of 20 μg/μL of Hoechst 33342 (e.g., dilute 14 μL of a 10 mg/mL stock solution into 7 mL) in PBS per plate. Mix the solution.
19. Remove 100 μL of medium from the inner wells and add 100 μL of stain to each well for a final concentration of 10 μg/μL Hoescht in 2% (w/v) paraformaldehyde. Wrap the plate in aluminum foil and let sit for at least 30 min at 21°C or for 8–48 h (usually overnight) at 4°C.
20. Using a high content imaging system (e.g., the Perkin Elmer Operetta), count the number of nuclei per plate to normalize the data to cell number as in Figure 2.
  a. The three risers in the Seahorse XF well should be blacked out to avoid complications with automated cell counting. As shown in Figure 2A, the XF well is divided into 31 sections, three of which contain the circular risers and are thus excluded.

**Figure 2:**
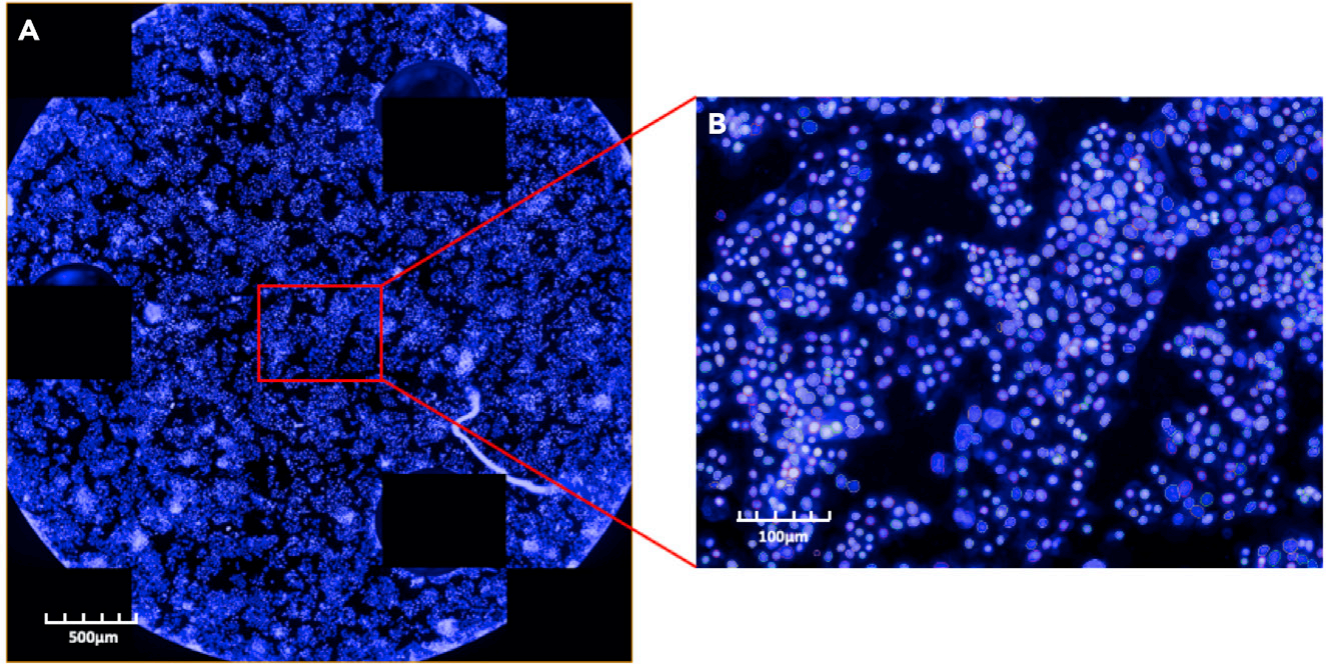
Example of automated cell counts Sample cell counts of HepG2 cells using the Perkin-Elmer Operetta plated at 2.5 × 10^4^ cells/well in Seahorse XF96 plates two days prior to the assay. (A) Computer-generated, full-well image of cell counts comprising the 31 fields imaged at 20× magnification to reconstitute the full plate. (B) Representative section showing identification of individual cells (nuclei outlined in various colors).
21. Normalize oxygen consumption rates from the permeabilized Seahorse XF data to pmol O_2_/min/1 × 10^3^ cells. This can be done either in the Seahorse XF Wave software using the normalization tab, or in spreadsheet software (e.g., Excel) after the assay is completed (Gotoh et al., 2021; Gu et al., 2021)
  a. For isolated mitochondria, the data is generally presented as pmol O_2_/min/μg mitochondrial protein.

## Expected outcomes

**Figure 3:**
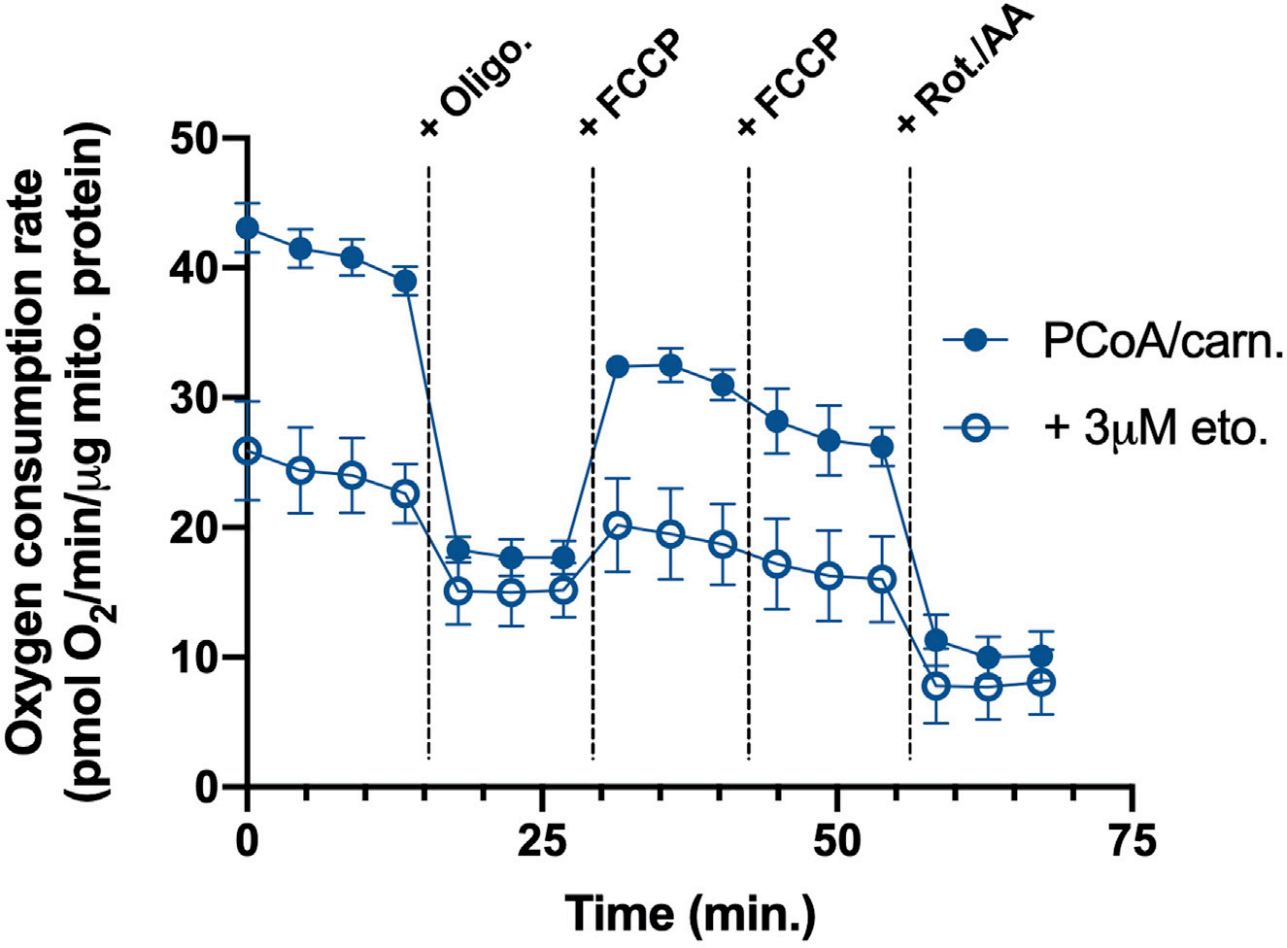
Sample respiratory trace for isolated liver mitochondria offered palmitoyl CoA with carnitine and malate ± 3 μM etomoxir Data are presented as mean ± SEM (n=5 technical replicates).

Figure 3 provides an example of results for respiration for either permeabilized cells or isolated mitochondria offered palmityol CoA with carnitine and malate. A robust rate of respiration is observed initially in the presence of excess ADP and substrate. The addition of oligomycin causes a sharp drop in respiration, as inhibition of the ATP synthase blocks consumption of the membrane potential and therefore slows respiration. Addition of the uncoupler FCCP restores respiration as membrane potential consumption stimulates electron transport chain activity. Finally, addition of the complex I inhibitor rotenone and the complex III inhibitor antimycin A block respiratory chain activity (Divakaruni et al., 2014).

**Figure 4:**
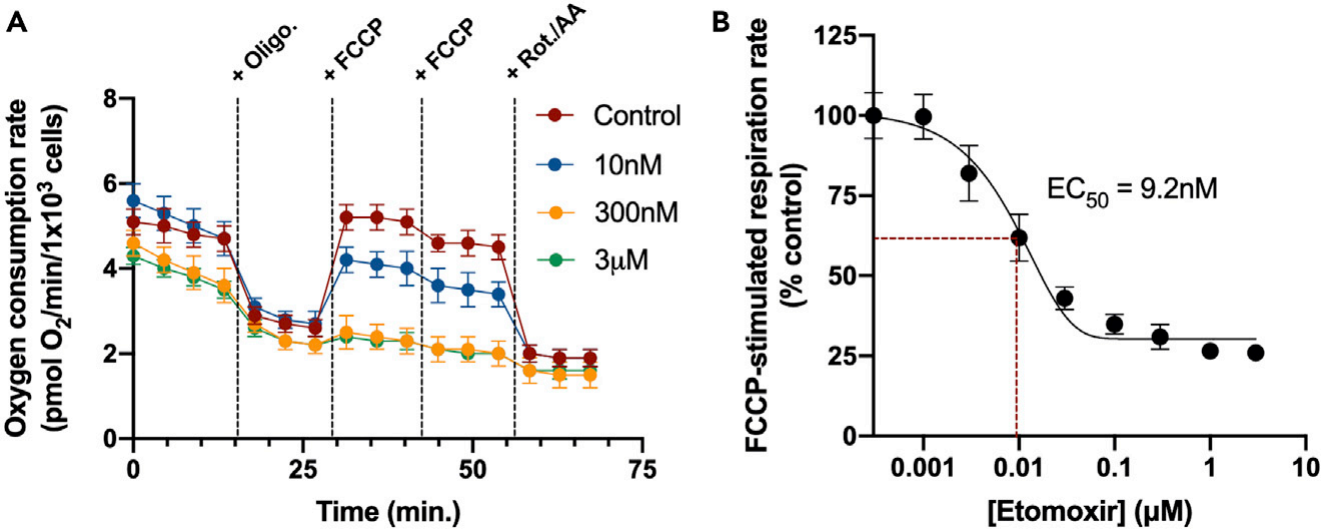
Etomoxir concentration-response curve in permeabilized cells (A) Representative respirometry trace in permeabilized HepG2 cells offered palmitoyl CoA and carnitine with malate and varying concentrations of etomoxir. (B) Concentration-response curve of FCCP-stimulated respiration in permeabilized HepG2 cells (as in A) in response to increasing concentrations of etomoxir. Red lines identify the EC_50_ value (9.2 nM) for etomoxir from this particular experiment. When not visible, error bars are obscured by the symbol. Data are presented as mean ± SEM (n=5 technical replicates).

**Figure 5:**
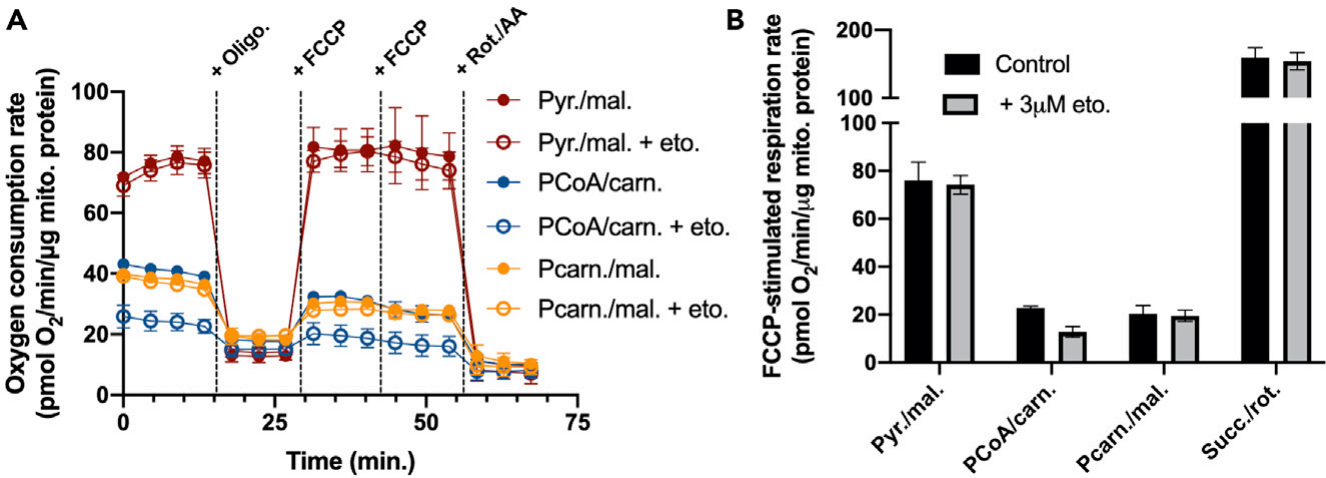
Etomoxir specificity in isolated mitochondria (A) Sample trace of respiration in isolated liver mitochondria offered (i) pyruvate with malate (Pyr./mal.), (ii) palmitoyl CoA and carnitine with malate (PCoA/carn.), or (iii) palmitoylcarnitine with malate (Pcarn./mal.) as respiratory substrates all ± 3 μM etomoxir. (B) Calculation of FCCP-stimulated respiration shows etomoxir at this concentration only affects palmitoyl CoA-driven respiration without affecting respiration driven by other substrates, thereby localizing the drug effect to CPT-1. Abbreviations are as before. Succ./rot., succinate/rotenone. Data are presented as mean ± SEM (n=6 technical replicates).

Importantly, the concepts of ‘basal respiration’ and ‘spare respiratory capacity’ often used to describe respirometry parameters in intact cells are not applicable for the reductionist systems described in this protocol. Whereas the basal respiratory rate largely reflects the rate of ATP turnover (i.e., work) in a live cell, excess ADP is given to permeabilized cells or isolated mitochondria in order to consume the membrane potential and support respiration. This explains why the rate of phosphorylating respiration is often similar to rates of uncoupled respiration in these reductionist systems. Figures 4 and 5 further demonstrate use of this protocol to use respiration driven by palmitoyl CoA and carnitine to assess alterations in CPT-1 activity, with the inhibitor etomoxir presented as a representative intervention (Divakaruni et al., 2018).

### Permeabilized cells

Figure 4 provides results for a concentration-response curve for the CPT-1 inhibitor etomoxir (Ceccarelli et al., 2011). To measure the potency of the compound, increasing concentrations of the drug were included with the assay medium (1 nM–10 μM in a semi-logarithmic concentration-response curve). Permeabilized HepG2 cells were offered palmitoyl CoA with carnitine and malate as substrate to directly measure respiration supported by CPT-1 (Figure 4A). In this system, etomoxir inhibited FCCP-stimulated respiration with nanomolar efficacy (EC_50_ = 9.2 nM) with a saturable response at sub-micromolar concentrations (Figure 4B).

### Isolated mitochondria

Although the experiment in Figure 4 shows etomoxir is a potent inhibitor of CPT-1, it does not demonstrate specificity, as inhibition of several enzymes other than CPT-1 could produce the same result. A standout feature of plate-based respirometry, however, is the ability to assess multiple oxidative pathways in a single experiment. In that vein, Figure 5 shows respiration in isolated mitochondria driven by palmitoyl CoA as well as other substrates that do not use CPT-1 and therefore are unaffected by the addition of 3 μM etomoxir.

## Quantification and statistical analysis

**Figure 6:**
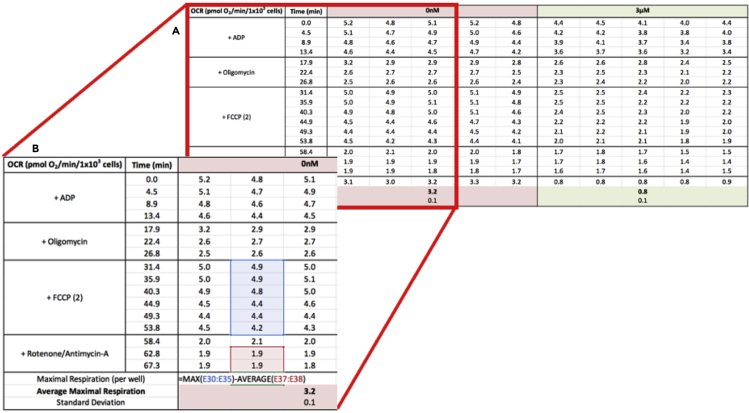
Sample spreadsheet calculations (A) Representative data for individual replicates. (B) Calculation of FCCP-stimulated, “maximal” respiratory rates corrected for background rates insensitive to rotenone and antimycin A.

Data analysis for permeabilized cells and isolated mitochondria is similar. The most useful parameters for this analysis are usually the FCCP-stimulated (“maximal”) respiration rate, phosphorylating respiration rate, and, in some cases, proton leak-linked respiration. Usually, the average of the two measurements prior to an injection are taken as the value for a particular parameter. The lone exception is the FCCP-stimulated rate, where the maximum value, irrespective of when it occurs during the measurement period, is used. It is essential that all values be adjusted for respiration insensitive to rotenone and antimycin A, which in these reductionist systems almost certainly represents measurement background. Excel formulas and sample calculations for a given well are shown below using the values from the second column of data in Figure 6.

1. Maximal, FCCP-stimulated respiration: MAX(FCCP) – Rot/AA
  = 4.9–1.9 = 3.0 pmol O_2_/min/1 × 10^3^ cells
2. Phosphorylating respiration: ADP – Oligo.
  = 4.5–2.65 = 2.85 pmol O_2_/min/1 × 10^3^ cells
3. Proton leak-linked respiration: Oligo. – Rot/AA.
  = 2.65–1.9 = 0.75 pmol O_2_/min/1 × 10^3^ cells

## Limitations

A major limitation of the assay is the inability to directly assess CPT-1 function. Oxygen consumption supported by CPT-1 is only an indirect measurement of enzyme activity, and requires the assumption that CPT-1 activity is entirely rate-controlling for palmitoyl CoA oxidation. It is well established that CPT-1 is a key rate-controlling enzyme for long-chain fatty acid oxidation (McGarry and Foster, 1980), though additional rate-controlling steps downstream of CPT-1 could limit the respiratory rate and lead to an incomplete assessment of direct CPT-1 activity.

An additional scientific limitation of the technique is the inability to measure CPT-1 activity under native conditions. While reductionist systems such as permeabilized cells and isolated mitochondria allow for the direct delivery of palmitoyl CoA and carnitine to mitochondria, stripping away the native cytoplasmic environment removes physiological regulatory factors. The technique only measures maximal capacities of substrates offered rather than native rates under basal conditions with various substrates present.

Some of the reagents or methods described may be cost-prohibitive for some labs, but alternative approaches are possible. Plant sterol glycosides, such as digitonin or saponin, can be used in lieu of perfringolysin O if they are properly titrated to find a concentration that permeabilizes the plasma membrane while keeping the mitochondrial inner membrane intact. Additionally, the method described uses the Seahorse XF Analyzer, though the work can be adapted to other platforms such as fluorescence or platinum-based electrode measurement systems (Doerrier et al., 2018; Will et al., 2007).

## Troubleshooting

In general, it is important to differentiate between problems with the instrumentation, reagents, or biological model. Examining the microplate at all stages of the assay under a light microscope to ensure a uniform monolayer, checking whether traces from individual wells show the same behavior within each group, and running multiple groups each with different substrates are all essential best practices and critical for troubleshooting.

### Problem 1

The mitochondrial isolation results in a poor yield or mitochondria with poor quality that do not respire with rates comparable to this protocol or other published data (Before you begin: steps 4–14).

### Potential solution

Several issues associated with the mitochondrial prep could result in suboptimal yield or quality. Some essential tips are listed below:

At every step of the isolation procedure, the sample material should be kept ice cold to avoid deterioration of the mitochondrial sample.

Remake all buffers, filter sterilize, and check the pH on the day of the assay at the appropriate temperature (the pH can change inversely with temperature). Ensure that all buffers are pre-chilled to 4°C prior to the experiment.

Work as quickly as possible from the time the animal is anesthetized (Before you begin: step 5) until collecting the pellet from the fast-speed spin (Before you begin: step 13). Working too slowly when removing the tissue from the animal or mincing/cleaning the tissue risks hypoxic damage. Prolonged waiting times or not keeping the sample appropriately cold between the slow- and fast-speed spins may result in mitochondrial damage from endogenous proteases in the homogenate.

Low yields could be due to insufficient homogenization. If using a hand-driven glass-on-glass homogenizer, try using a drill-driven Teflon-on-glass setup as shown in this protocol.

Poor quality could be due to over-homogenization. This will be characterized by a large light, “fluffy” layer, and perhaps mitochondria with higher-than-average proton leak-linked respiration. Two (or perhaps three) pulses with a drill-driven Teflon-on-glass setup should be sufficient to fully disrupt the liver from a single mouse.

It will likely require a few repetitions to be able to isolate mitochondria with the combination of speed and attention-to-detail necessary for a quality isolation.

### Problem 2

Rates are low and do not respond to mitochondrial effectors (step-by-step method details: steps 6–10 or 11–16).

### Potential solution

This could be due to several issues, some of which can be addressed with the trouble-shooting information below:

Sufficient material may not be on the microplate. Check whether cells or mitochondria remain in a confluent monolayer on the microplate with light microscopy. It may be that permeabilized cells are lifting off the microplate during the washing steps. This can be addressed with optimizing the cell density for this specific assay, being more gentle when washing the plate, or using a plate coating such as poly-D-lysine. Cells could also be acutely spun down onto the plate with Cell-Tak, as is done for cells grown in suspension. If this occurs with isolated mitochondria, this could be due to improper protein determination or uneven mixing of the mitochondria during dilution and plating. Looking at data from each well individually (use the dropdown menu in Wave software to switch from “group” view to individual “well” view) can be useful to help determine whether the problem is shared across the entire plate or only within specific wells.

The pH of the medium may be incorrect. Switch the display to ECAR and click the “Level” button to determine whether the effectors or MAS were appropriately pH’d.

It may be that isolated mitochondria were damaged during the isolated procedure. See problem 1 for troubleshooting tips for isolated mitochondria.

It may be that cells/mitochondria were inadvertently plated in the background wells or background wells were inappropriately assigned. It is possible to uncheck the “Background correction” box to view general trends during the assay.

### Problem 3

Rates are initially high and stable but progressively drop during the course of the assay or become increasingly variable (step-by-step method details: steps 6–10 or 11–16).

### Potential solution

This could be due to several issues, some of which can be addressed with the trouble-shooting information below:

If using permeabilized cells, this could be due to cells detaching from the microplate during the course of the assay. See problem #2 for how to troubleshoot cells detaching from the plate.

It may be that injected compounds, particularly rotenone and antimycin A, are leaking into the ports prematurely. When loading the ports, take care to maintain steady pressure so that injectate leaves the pipet tip smoothly, rather than in separate droplets. Do not eject the solution too quickly or vigorously to prevent leaking, and confirm the absence of bubbles in the pipet tips after drawing up injectate for loading.

It is also possible that the instrument and reagents are not the issue, but rather a very low expression of CPT-1 in the cells or mitochondria under investigation. High initial rates can reflect use of residual or endogenous substrates. Running controls during the assay such as pyruvate with malate or succinate with rotenone is often helpful to differentiate between an issue with instrumentation, reagents, or model system.

Minor changes in the initial rate that eventually stabilize may also reflect the plate warming to 37°C, as the oxygen-sensitive fluorophore that was previously calibrated at 37°C may behave differently when not at the appropriate temperature.

### Problem 4

The injector ports are not injecting properly/evenly (step-by-step method details: step 3).

### Potential solution

This could be due to inadvertently not filling all the ports properly, leaving a path for the pulse of air to escape without entirely injecting the contents of the port. Ensure the medium makes a full seal of the port, and that BSA is absent from the injectate medium to avoid issues with medium viscosity. See solutions to Problem #3 for additional tips for properly loading the injector ports. This issue also may also reflect improper injection from residue buildup inside the instrument, and requires a service call to clean the inner workings of the instrument.

### Problem 5

The concentration of mitochondrial effectors (e.g., oligomycin, FCCP, rotenone/antimycin A) needs to be adjusted (step-by-step method details: step 2).

### Potential solution

Oligomycin, rotenone, and antimycin A are all used at saturating concentrations in XF analysis experiments and are orders of magnitude above their respective EC_50_. Any titrations with these compounds should determine the lowest concentration required to give stable respiratory inhibition after compound addition. The concentrations provided in this protocol should be appropriate for most types of permeabilized cells or isolated mitochondria. When using high concentrations of BSA or working with lipid-rich cells such as 3T3-L1 adipocytes, it may be necessary to increase the measurement time in order to reach stable, steady-state readings after injecting oligomycin.

The appropriate amount of FCCP will be dependent on the cell type, BSA concentration, substrates and co-substrates offered, and prior drug treatment or genetic modification. For this reason, we advocate using two pulses of FCCP through ports B & C to cover a broader range of concentrations over a single assay. If the concentration needs to be titrated or adjusted, the researcher should determine the concentration that yields a maximal yet relatively stable rate (i.e., the rate does not immediately peak and sharply trail down during subsequent measurements).

## Resource availability

### Lead contact

Further information and requests for resources and reagents should be directed to and will be fulfilled by the lead contact, Ajit Divakaruni (adivakaruni@mednet.ucla.edu).

### Materials availability

This study did not generate new, unique reagents.

### Data and code availability

The data supporting the current study have not been deposited in a public depository as they were generated only to serve as representative examples, and are available from the corresponding author upon request.
